# Effects of SOCS1-overexpressing dendritic cells on Th17- and Treg-related cytokines in COPD mice

**DOI:** 10.1186/s12890-022-01931-1

**Published:** 2022-04-15

**Authors:** Shi-xia Liao, Jie Chen, Lan-Ying Zhang, Jing Zhang, Peng-Peng Sun, Yao Ou-Yang

**Affiliations:** 1 Department of Respiratory Medicine, Affiliated Hospital of Zunyi Medical University, Guizhou, 563003 China; 2 Department of Osteopathy, Affiliated Hospital of Zunyi Medical University, Guizhou, 563003 China

**Keywords:** Chronic obstructive pulmonary disease, Dendritic cells, SOCS1, Th17, Treg

## Abstract

**Background:**

In this study, we established a chronic obstructive pulmonary disease (COPD) model by stimulating mice with cigarette smoke, and observed the effects of dendritic cells (DCs) overexpressing SOCS1 on Th17, Treg and other related cytokines in peripheral blood, bronchoalveolar lavage fluid and lung tissues of COPD mice.

**Methods:**

After successfully transfecting DCs with overexpressing SOCS1 (DC-SOCS1), the mice were injected with DC-SOCS1 (1 × 10^6^), DC-SOCS1 (2 × 10^6^) and immature DCs (1 × 10^6^) via tail vein on days 1 and 7 of COPD fumigation modeling. After day 28 of modeling, the peripheral blood, BALF and lung tissue samples were extracted from the mice, and the changes of DCs, Th17 and Treg cells and related cytokines were detected by immunohistochemistry, immunofluorescence, HE staining, flow cytometry and ELISA.

**Results:**

The results showed that DC-SOCS1 was able to reduce the secretion of pro-inflammatory factors and increase the anti-inflammatory factors in the COPD mice, and the effect of high concentration (2 × 10^6^ DC-SOCS1) was better than low concentration (1 × 10^6^ DC-SOCS1). Moreover, the intervention effect was significant on day 1 compared with day 7. In the mice injected with DC-SOCS1, the expression of CD83, IL-4, Foxp3, and CCR6 was increased on day 1 than those on day 7, while IL-17 and IFN-γ was decreased.

**Conclusions:**

Intervention of COPD mice with high concentrations of DCs-SOCS1 reduced pro-inflammatory factor secretion and attenuated the inflammatory response in COPD.

*Trial registration* Not applicable.

**Supplementary Information:**

The online version contains supplementary material available at 10.1186/s12890-022-01931-1.

## Background

Chronic obstructive pulmonary disease (COPD) is a chronic inflammatory disease of the airways characterized by incomplete reversibility and progressive airflow limitation, associated with an enhanced chronic inflammatory response of the airways and lungs to toxic particles or gases [1, 2]. The incidence and mortality rates of COPD have continued to increase in recent years [3].

In addition, it has been found that COPD may be an immune disease caused by smoking and its pathogenesis may be related to the deregulation of the autoimmune system [4, 5]. T lymphocytes play an important role in the immune regulatory mechanism of COPD, mainly by mediating airway inflammation or immune response, which requires the participation of dendritic cells (DCs) [6, 7]. DCs are the most important specialized antigen presenting cells (APCs) in the body, which can take up, process and present antigens, stimulate the proliferation and activation of initial type T cells, and are central to the initiation, regulation and maintenance of specific immune responses [8, 9]. As a recent study, the role of DCs in immune-related diseases has become a hot topic. Depending on their maturation status, DCs can be divided into imDCs and mDCs. The imDCs are typically tolerogenic and induce immune tolerance by regulating T-cell responses to keep the body in a balanced and healthy state [10]. The suppressor of cytokine signaling (SOCS) 1 is a negative regulator of the signal transducer and activator of transcription (STAT) pathway of Janus kinase (JAK), which is involved in the differentiation, activation, and maturation of DCs, and the overexpression of SOCS1 can maintain DCs in the state of imDCs [11, 12]. In recent studies, SOCS1 was found to be involved in the pathogenesis of COPD and related to the time of COPD onset [11]. However, the effects of overexpressed SOCS1-regulated DCs on T helper 17 (Th17), regulatory T cells (Treg) and other related cytokines in the pathogenesis of COPD have not been clarified.

Therefore, this study was conducted to observe the effects of DCs overexpressing SOCS1 on Th17, Treg and other related cytokines in peripheral blood, bronchoalveolar lavage fluid (BALF) and lung tissues of COPD mice, and provided a new perspective for COPD treatment.

## Materials and methods

### Cell culture

A number of healthy 6–8 weeks old C57BL/6 mice, purchased from the Animal Experiment Center of the Third Military Medical University. Mice were anesthetized and disinfected with 75% ethanol, then the femur and tibia were taken under aseptic conditions and washed twice with phosphate buffered saline (PBS) solution. The epiphysis was removed to expose the bone marrow cavity, and the bone marrow was flushed with PBS until the bone marrow cavity turned white. The flushed bone marrow was made into single cell suspension and centrifuged at 1000 r/min for 5 min (min), and the supernatant was discarded. Resuspended the cell precipitate with PBS. The cell suspension was slowly added into a 15 ml centrifuge tube pre-filled with 5 ml lymphocyte separation solution, centrifuged at 2000 rpm for 20 min, and the solution was divided into 5 layers after centrifugation, and the intermediate bone marrow stromal cell layer was aspirated and washed 3 times with PBS. The cells were inoculated with DC cell growth medium containing 10% fetal bovine serum and penicillin at a concentration of 10^6^/ml cells in 6-well plates and incubated at 37 ℃ in a 5% CO_2_ incubator. Cells were plastered for about 1–2 days (d) and maturation was induced by adding LPS (100 ng/μl) on day 7 of culture.

All animal experiments were carried out in accordance with the principles of the guidelines described in the Care and Use of Animals, and had been approved by the animal experimentation committee of the Zunyi Medical University.

### Flow cytometry (FCM) assay

CD80 and CD83 antibodies were used to identify DCs. The cells were collected after 24 h of Lipopolysaccharide (LPS) induction, then centrifuged at 1000 rpm for 5 min to precipitate the cells, and the supernatant was discarded. Resuspended the cells. Then, 50 μl diluted antibodies (antibodies diluted to the appropriate concentration with Staining Buffer 2–0.03 μg/10^6^ cells) were added to each flow assay tube; 50 μl Staining Buffer was added to the blank or isotype control tube. In each tube, 50 μl cell suspension (approximately 10^6^ cells) was added and gently mixed. After incubation for 30 min in an ice bath, the cells were incubated with Staining Buffer (2 ml per tube and 200 μl per well) and centrifuged at 1000 rpm for 5 min at 4 °C. The supernatant was discarded and washed three times. 100 μl of the cells were resuspended and detected by FCM.

### Electron microscopy

After the cells were fixed, dehydrated and dried, the samples were placed on a sample stage approximately 10–15 cm away from the evaporation source and subjected to conductive treatment to observe the morphology of the induced mDCs under electron microscopy.

### Construction of SOCS1 gene overexpression lentiviral vector

The SOCS1 gene (Gene ID: 12703) was searched on the NCBI website, and NM_001271603.1 transcript was chosen for sequence synthesis. The Kozak sequence and EcoRI digested site were added at the 5′ end, and the BamHI site was added at the 3′ end. The pLVX-IRES-ZsGreen1 vector (Fig. 2a) was digested with EcoRI and BamHI. The purified synthetic product was ligated to the linearized vector, and the ligated product was transformed into bacterial receptor cells. The grown clones were first identified by enzymatic cleavage to demonstrate that the target gene had been targeted into the target vector. After sequencing and analyzing the positive clones, the constructed lentiviral overexpression plasmid vector was subjected to high-purity endotoxin-free extraction and then co-transfected with the lentiviral packaging vector in 293T cells. The supernatant was collected, purified and concentrated to be the high titer pLVX-SOCS1-IRES-ZsGreen1 lentivirus. The virus titer was detected by multiplicative dilution method after infection of 293T cells. The lentiviral packaging vector is a four-plasmid system consisting of pLVX-IRES-ZsGreen1, PG-p1-VSVG, PG-P2-REV, and PG-P3-RRE (Fig. 2b–d). Of these, pLVX-IRES-ZsGreen1 is able to express green fluorescent protein (GFP), and PG-p1-VSVG, PG-P2-REV, and PG-P3-RRE contain components essential for viral packaging. The results of pLVX-SOCS1-IRES-ZsGreen and negative control lentivirus titer assay were presented in Fig. 2e.

### Lentiviral infection of DCs

The primary DCs were isolated and cultured, and lentiviral infection was performed on the fifth day after isolation. The virus solution was aspirated and added to the cells, along with 5 μg/ml of polybrene co-transfection reagent to improve the infection efficiency. Then, the 24-well plates were incubated at 37 °C and the cell status was observed after 8–12 h (h). After 24 h, the medium was replaced with fresh medium and observed under fluorescence microscope after 48 h of infection. DCs with green fluorescence were those infected with lentivirus overexpressing on SOCS1 gene. Meanwhile, samples were collected for further study.

### Quantitative real-time PCR (qRT-PCR)

qRT-PCR for detecting transfection effects after lentivirus infection of DCs. SYBR Green I is a fluorescent dye that binds to double-stranded DNA and emits light, allowing detection of the amount of double-stranded DNA present in the PCR system based on the fluorescent signal. The RNA was first reverse transcribed into cDNA using random primers, and then specific primers and SYBR Green I fluorescent dye were designed for fluorescent quantitative PCR detection. The primer sequences were: SOCS1 (ID: 12703), mSOCS1F: CGCCTGCGGCTTCTATTG, mSOCS1R: CCCGAAGCCATCTTCACG, m actin f: GAGACCTTCAACACCCCAGC, m actin r. ATGTCACGCACGATTTCCC.

### Western blot

Western blot (WB) was used to detect the transfection effect after lentivirus infection of DCs. The cultured DCs infected with lentivirus were lysed with RIPA lysis buffer, centrifuged at 12,000 rpm, 4 °C for 15 min, and the supernatant was collected. Then the protein concentration was determined by BCA protein assay kit. The protein was separated on 10% sodium dodecyl sulfate polyacrylamide gel electrophoresis and subsequently transferred to polyvinylidene fluoride membranes (Bio-Rad), which were subsequently blocked with Tris Buffered saline Tween (TBST) solution containing 5% skim milk at room temperature for 2 h. The corresponding primary antibody (SOCS1: Abcam, anti-rabbit) was diluted to a certain concentration (1:500) with the blocking solution, and the final concentration of the internal reference primary antibody was 1:1000, and then incubated overnight at 4 °C. After 3 washes with TBST, they were incubated with secondary antibodies (Sigma, goat-anti-rabbit, 1:1000) at room temperature for 1 h. Finally, samples were incubated with the ECL-enhanced chemiluminescence detection kit (Thermo Scientific Pierce™) and OD values were calculated with ImageJ pro plus.

### COPD modeling in C57BL/6 mice and DCs reinfusion

Thirty-eight 8-week-old male SPF-grade C57BL/6 mice, weighing 20–25 g (Chongqing Tengxin Biotechnology Co., Ltd.) were obtained. The 35 mice were randomly assigned into 7 groups (B-H groups), and the 7 groups were housed in identical environments (Table 1). The animals in each group were placed in a homemade smokebox (45 cm × 30 cm × 30 cm) and smoked passively 3 cigarettes/times, 1 h/times, 4 times/day (Fig. 3a–c). The time points were 9:30, 11:00, 14:00, 15:30, 7 days, and 28 days of smoking. The general condition of each group was observed, and the body mass of each group was weighed and recorded weekly before and after fumigation to dynamically observe the effect of fumigation on the body mass of mice.

**Table 1:** Grouping of COPD modeling in C57BL/6 mice and cell reinfusion

Group	Number	Operation
A: Normal group	N = 3	No fumigation
B: NC group	N = 5	2 × 10^6^ cells/ml, 0.2 ml/10 g, injected on day 1 of fumigation
C: DC-SOCS1 I group	N = 5	1 × 10^6^ cells/ml, 0.1 ml/10 g, injected on day 1 fumigation
D: DC-SOCS1 II group	N = 5	2 × 10^6^ cells/ml, 0.1 ml/10 g, injected on day 1 fumigation
E: imDCs I group	N = 5	1 × 10^6^ cells/ml, 0.1 ml/10 g, injected on day 1 fumigation
F: DC-SOCS1 III group	N = 5	1 × 10^6^ cells/ml, 0.1 ml/10 g, injected on day 7 fumigation
G: DC-SOCS1 IV group	N = 5	2 × 10^6^ cells/ml, 0.1 ml/10 g, injected on day 7 fumigation
H: imDCs II group	N = 5	1 × 10^6^ cells/ml, 0.1 ml/10 g, injected on day 7 fumigation

### Tissue harvest

After 28 days of modeling, the peripheral blood was collected from the eyeballs for FCM assay. The trachea was exposed by cutting the skin in the middle of the neck, and the trachea and alveoli were irrigated with saline (0.6 ml/time, for 3 times). The BALF was collected and centrifuged (1500 r/min, 10 min, 4 °C), and the supernatant was used for enzyme linked immunosorbent (ELISA) assay and the BALF precipitate was taken for FCM assay. The abdominal cavity was opened, and fresh liver, spleen and lung tissues were washed in cold PBS, and part of the lung tissues were taken for FCM assay, while the rest of the tissues were stored at − 80 °C for ELISA assay. The tissues were fixed in 4% paraformaldehyde for more than 24 h for immunohistochemistry, immunofluorescence and hematoxylin–eosin staining.

### Hematoxylin–eosin (HE) staining

Tissues fixed in 4% paraformaldehyde fixative for 24 h were paraffin embedded, sectioned, stained with hematoxylin stain and eosin stain, and sealed with neutral resin and observed under the microscope. The nucleus became blue, while the cytoplasm became red or pink.

### Immunohistochemistry (IHC)

Tissues fixed in 4% paraformaldehyde fixative for 24 h were subjected to IHC experiments after paraffin embedding, sectioning, and antigen thermal repair, and the changes of IL-17, IL-4, CD83, CCR6, Foxp3 and INF-γ factor levels were detected in each group. The tissues were washed with PBS and then closed with goat serum at room temperature for 1 h. Samples were then incubated with primary antibodies overnight at 4 °C (IL-17A: dilution ratio 1:200; CCR6: 1:100; CD83: 1:100; Foxp3: 1:200; IL-4: 1:100; IFN-γ: 1:100). Following PBS wash, (HRP-labeled) secondary antibodies working solution was incubated at room temperature for 2 h. After DAB color development at room temperature for 1–10 min (avoid light), distilled water wash, hematoxylin re-staining for 5 min. Then wash back to blue with tap water, gradient alcohol dehydration (75%, 85%, 95%, 100% for 3 min each), xylene transparent 2 times for 3 min. Lastly, the sections were sealed with neutral resin and observed under the microscope.

### Immunofluorescence (IF)

Paraffin sections of the samples were dewaxed, rehydrated, washed with double-distilled water, and washed with PBS, then incubated with DAPI (4′,6-diamidino-2-phenylindole) for 15 min protected from light. Finally, anti-fluorescence quenching agent was added to seal the slides and the changes of each group was observed under fluorescence microscope.

### FCM detection of DCs, Th17 and Tregs

For Th17, individual nucleated cells were resuspended in RPMI 1640 (10% heat-inactivated FBS) and counted by Tissue Blue staining; the cell concentration was adjusted to 2 × 106 cells/ml, 500 μl. Cells were treated with PMA/Ionomycin Mixture (250 ×) for 5–18 h in each group, along with protein transport inhibitor (BFA/Monensin Mixture (250 ×).

For Treg, the 100 µl cell suspension was added to each tube, with a cell count of approximately 1 × 106 cells. The surface antigens were labeled according to the cell surface antigen staining method, and the appropriate amount of CD4 and CD25 antibodies were added according to the instructions and incubated for 30 min at room temperature without light.

Then, the cells were washed with pre-chilled PBS and the supernatant was discarded after centrifugation. Then the cells were resuspended by adding 1 ml of Fixation/Permeabilization solution and incubated for 30 min at room temperature under dark conditions. Subsequently, 1 ml Permeabilization Buffer (diluted 1:9 in deionized water) was added, and supernatant was discarded after centrifugation. Cells were resuspended with 100ul Permeabilization Buffer, added IL-17A (for Th17)and Foxp3 (for Treg) antibody, and incubated for 30 min at room temperature without light. Finally, it was washed with flow staining buffer, centrifuged at 300 g for 5 min, resuspended to 500ul with flow staining buffer, and detect on the machine.

For DCs, the 100 µl cell suspension was added to each tube, with a cell count of approximately 1 × 10^6^ cells. The surface antigens were labeled according to the cell surface antigen staining method, and the appropriate amount of CD80 and CD83 (CCR5 and CCR6 for imDCs) antibodies were added according to the instructions and incubated for 30 min at room temperature without light. The cells were washed with pre-chilled PBS and the supernatant was discarded after centrifugation. Then, the cells were washed with flow staining buffer, centrifuged at 300 g for 5 min, resuspended to 500ul with flow staining buffer, and detect on the machine.

### ELISA detection of Th17, Tregs, Th1 and Th2

The ELISA kit was used to determine the levels of interleukin (IL)-17, IL-21, IL-23, IL-10, transforming growth factor β (TGF-β), interferon-gamma (IFN-γ), IL-12, IL-4 and IL-5 in BALF supernatant and lung tissue samples in COPD mice. The kit applied double antibody sandwich method for the determination. The microtiters were coated with the purified mouse IL-17/IL-21/IL-23/IL-12/IL-10/IFN-γ/IL-4/TGF-β/IL-5 antibodies to make solid phase antibodies, and IL-17/IL-21/IL-23/IL-12/IL-10/IFN-γ/IL-4/TGF-β/IL-5 were added sequentially to the microplates. Then, it was conjugated with HRP-labeled IL-17/IL-21/IL-23/IL-12/IL-10/IFN-γ/IL-4/TGF-β/IL-5 antibodies to form antibody-antigen-enzyme-labeled antibody complexes, which were thoroughly washed and color developed by adding the substrate TMB. TMB was converted to blue by HRP enzyme and finally to yellow by acid. In addition, the shade of color was positively correlated with IL-17/IL-21/IL-23/IL-12/IL-10/IFN-γ/IL-4/TGF-β/IL-5 in the samples. The optical density (OD) value was measured at 450 m wavelength by microplate reader, and the concentration of mouse IL-17/IL-21/IL-23/IL-12/IL-10/IFN-γ/IL-4/TGF-β/IL-5 in the samples was calculated by the standard curve.

### Statistical analysis

Statistical analysis was performed using SPSS version 26.0, and the quantitative data were analyzed by one way ANOVA and shown as mean ± standard deviation. *P* < 0.05 was considered as statistically significant.

## Results

### Cell culture and identification of DCs

**Fig. 1:**
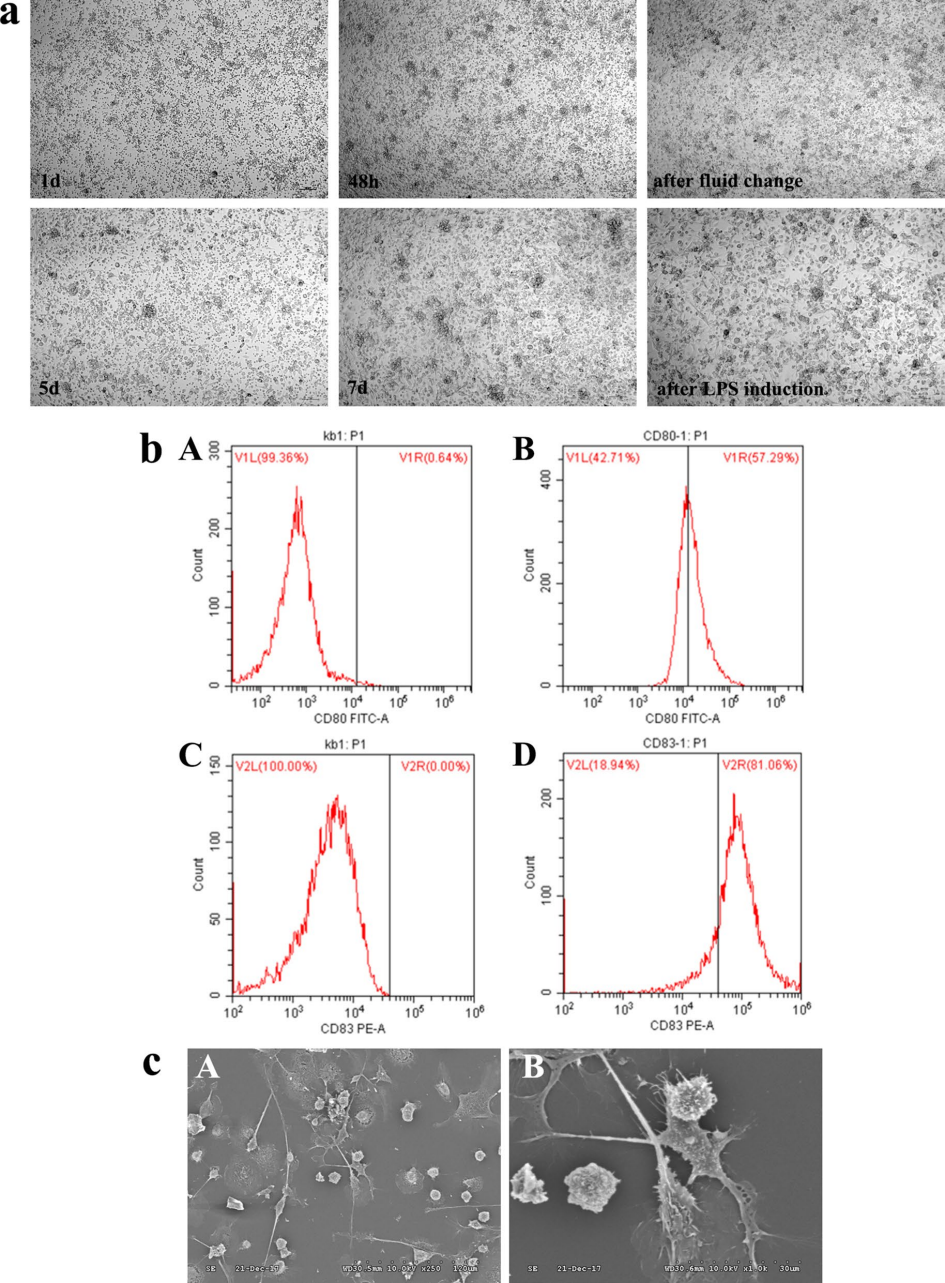
Cell culture and identification of DCs. **a** Observation of DCs cell culture status at each time point. **b** Identification of DCs culture results by flow cytometry (A. Isotype controls; B. CD80^+^DCs; C. Isotype controls; D. CD83^+^DCs). **c** Observation of DCs under electron microscope (A. 250X; B. 1000X)

The growth status of DCs in culture for 1 days, 48 h, after fluid change, 5 days, 7 days and after LPS induction was shown in Fig. 1a. Identification of DCs monolabeled with CD80 and CD83 antibodies was performed by FCM, which showed a significantly higher number of DCs and higher fluorescence signal intensity compared with the blank control, suggesting that DCs were successfully cultured (Fig. 1b). In addition, induced mDCs were also observed under electron microscopy (Fig. 1c).

### Transfection effect results of lentivirus infection of DCs

**Fig. 2:**
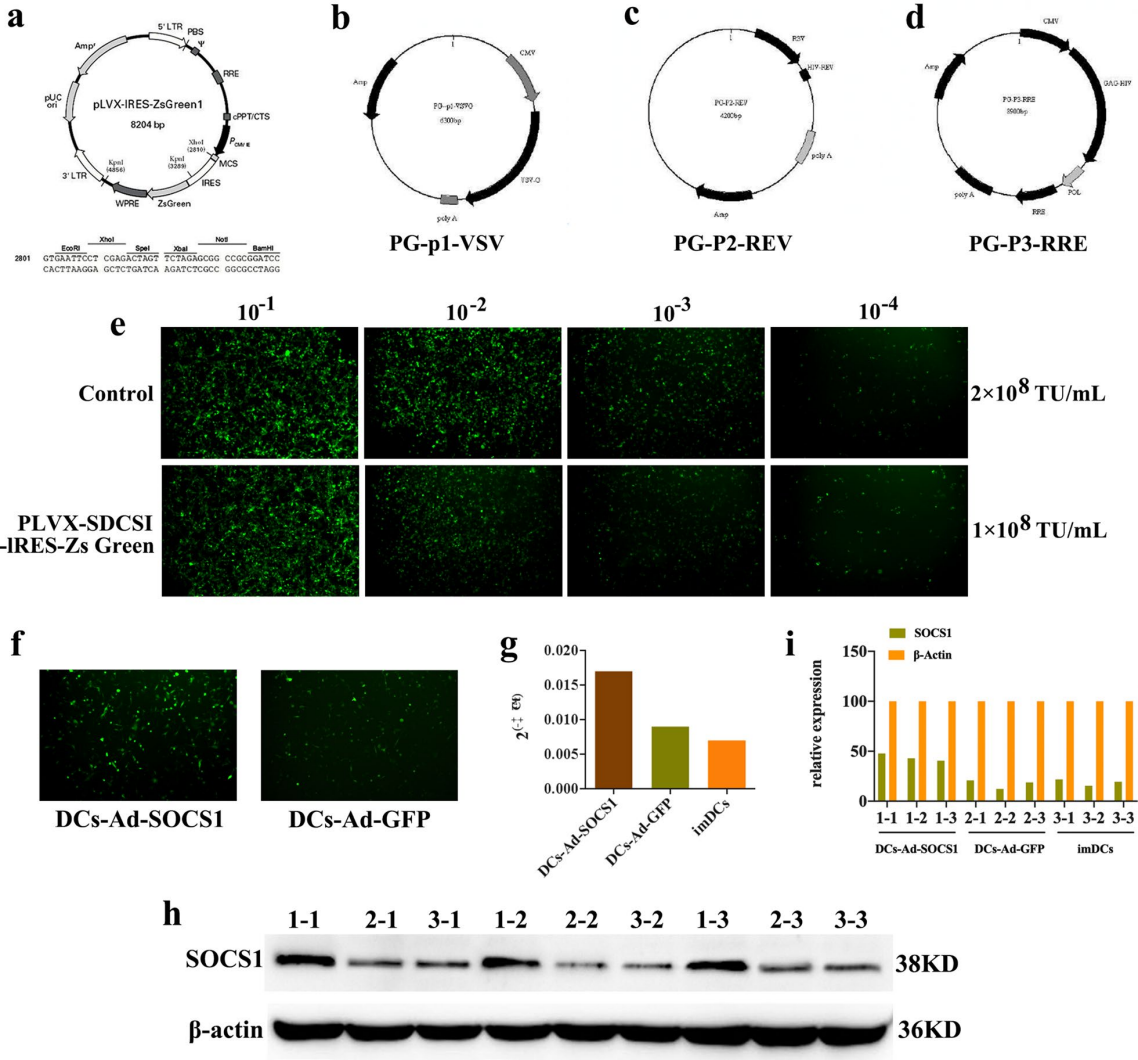
SOCS1 gene overexpression lentivirus transfection of DCs. **a** pLVX-IRES-ZsGreen1 vector. **b** PG-p1-VSVG. **c** PG-P2-REV. **d** PG-P3-RRE. **e** pLVX-SOCS1-IRES-ZsGreen and negative control lentivirus titer assay results. **f** Fluorescence microscopic observation of SOCS1 gene overexpression transfected DCs (DCs-Ad-SOCS1 group) and empty GFP lentivirus transfected DCs (DCs-Ad-GFP group) at 5 days of incubation. **g** DNA content of DCs-Ad-SOCS1, DCs-Ad-GFP, and imDCs groups. **h** WB bands. **i** WB grayscale analysis

The states of DCs transfected with overexpressed SOCS1 gene (DCs-Ad-SOCS1) and with GFP lentivirus no-load for 5 days (DCs-Ad-GFP) was observed under the fluorescence microscope as shown in Fig. 2f, with green fluorescence was the target cells. In addition, the amount of double-stranded DNA present in the PCR system could be detected based on the fluorescence signal intensity of SYBR Green I. The qPCR results showed that the amount of DNA in the DCs-Ad-SOCS1 group was significantly higher compared with the DCs-Ad-GFP and imDCs groups (control group) (Fig. 2g). WB showed that the expression of SOCS1 was higher in the DCs-Ad-SOCS1 group, indicating that the SOCS1 gene had been successfully transfected into DCs (Fig. 2h, i).

### Successful construction of COPD model and changes of DCs in lung tissue

**Fig. 3:**
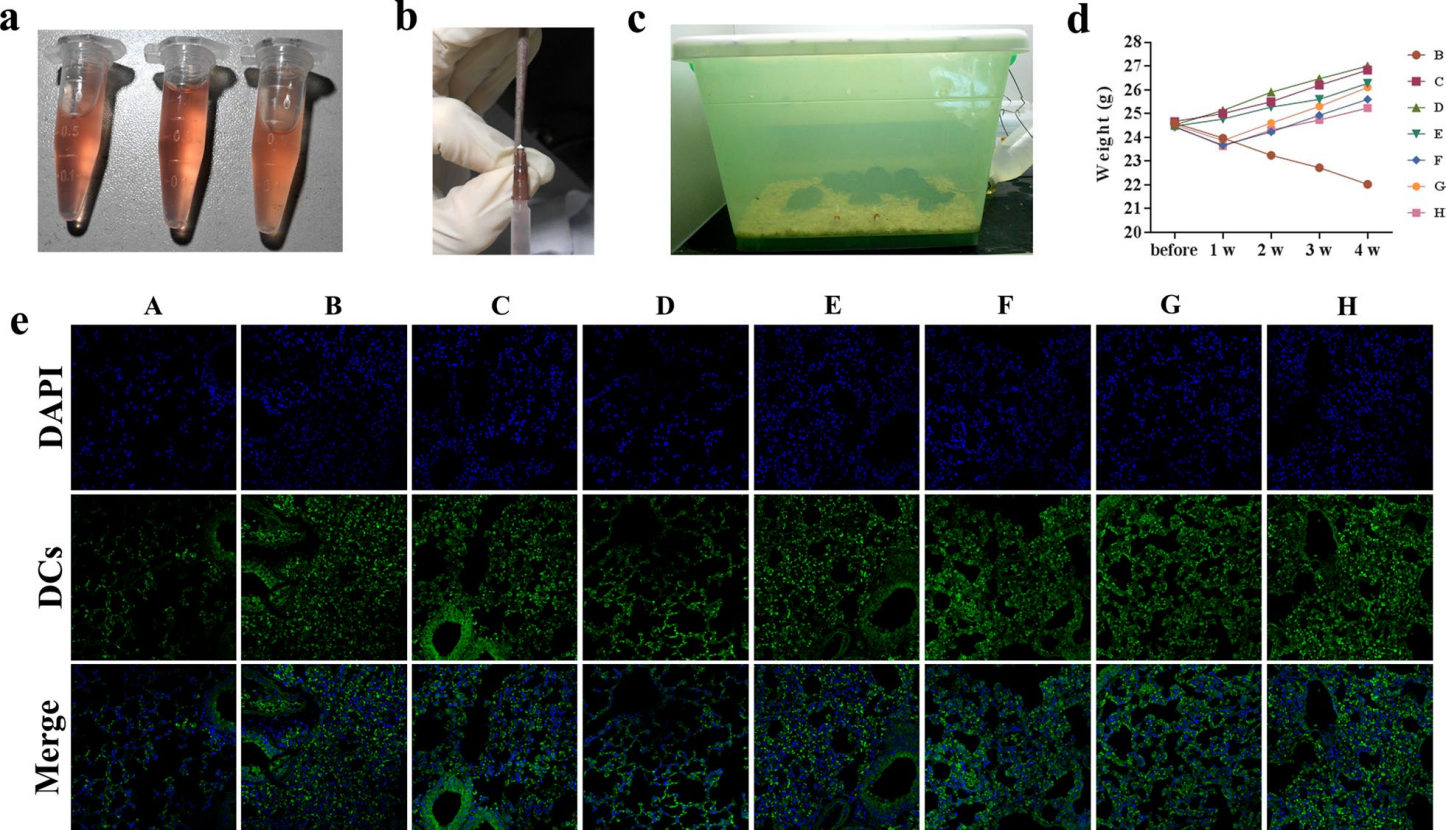
Establishment of COPD mice model. **a** Successfully transfected DC-SOCS1 cells. **b** DC-SOCS1 or imDCs were injected via tail vein. **c** Mice were smoked with cigarettes for COPD modeling. **d** Body weight changes of mice in each group after modeling. **e** Immunofluorescence observation of DC cell changes in lung tissue. (A. Normal group; B. NC group (DCs-Ad-GFP group); C. DC-SOCS1 I group; D. DC-SOCS1 II group; E. imDCs I group; F. DC-SOCS1 III group; G. DC-SOCS1 IV group; H. imDCs II group.)

The weekly changes in body mass of mice in each group before and after fumigation were presented in Fig. 3d. The B group, empty GFP lentivirus transfected DCs, showed a continuous decrease in body mass after fumigation. Groups C, D and E were treated with DC-SOCS1 or imDCs reinfusion on 1d of fumigation, and mice in all three groups had a continuous increase in body mass after fumigation. Groups F, G and H received DC-SOCS1 or imDCs reinfusion on the 7th day of fumigation, and the body mass of mice in the three groups decreased at 1 week after fumigation but continued to increase from the 2nd week, which meant that the body mass decreased before receiving the reinfusion and increased after the reinfusion treatment. That was to say, the body mass of mice in D and G groups that received DC-SOSC1 2 × 10^6^ reinfusion was greater than that of C and F groups that received DC-SOCS1 1 × 10^6^ reinfusion. Similarly, the body mass increase of mice in E group, which received imDCs reinfusion 1 day after fumigation, was greater than that in H group, which received imDCs reinfusion 7 d after fumigation. In addition, by immunofluorescence observation of lung tissue DCs, we found that compared with A group, there was a significant increase in DCs in B, E and H groups, more growth in C and F groups, a slight increase in G group and no significant change in D group (Fig. 3e).

### Content of DCs, Th17 and Tregs in peripheral blood, BALF sediment, and lung tissue

The content of mDCs (CD80^+^CD83^+^), imDCs (CCR5^+^CCR6^+^), Th17 (CD4^+^IL-17A^+^), and Treg (CD4^+^CD25^+^Foxp3^+^) in peripheral blood, lung tissue, and BALF sediment were determined using FCM and expressed as percentage (%) (Table 2).

**Table 2:** Content of mDCs, imDCs, Th17 and Treg in the peripheral blood, BALF sediment and lung tissue

Groups	mDCs	imDCs	Th17	Treg
In peripheral blood, F value	115.742	2.370	3.636	21.848
B group, %	14.25 ± 0.50	1.27 ± 0.09	2.16 ± 0.04	2.83 ± 1.03^#^
C group, %	9.14 ± 0.84	2.44 ± 1.12	2.05 ± 0.03	13.39 ± 1.55
D group, %	3.29 ± 0.21^*$%^	4.46 ± 0.23^*^	2.05 ± 0.09	19.47 ± 0.44
E group, %	11.37 ± 0.15	2.28 ± 0.99	1.97 ± 0.06	6.26 ± 0.29^#^
F group, %	8.98 ± 0.19^$^	2.44 ± 1.11	1.73 ± 0.15	8.04 ± 2.64
G group, %	5.13 ± 0.28^*$%^	2.75 ± 1.34	1.70 ± 0.01^@^	15.59 ± 3.34
H group, %	13.94 ± 0.98	1.95 ± 0.62	2.07 ± 0.29	6.26 ± 1.04^#^
In BALF sediment, F value	45.796	154.898	6.578	16.365
B group, %	4.95 ± 0.51	4.92 ± 0.18	3.68 ± 0.63	3.80 ± 0.09
C group, %	3.01 ± 0.02	6.69 ± 0.35	1.16 ± 0.11	4.69 ± 0.13
D group, %	1.98 ± 0.11	12.92 ± 0.01^*^	0.77 ± 0.08	5.54 ± 0.45
E group, %	3.58 ± 0.09^#&^	5.95 ± 0.03^#^	1.82 ± 0.55	4.39 ± 0.18
F group, %	3.32 ± 0.11^#^	6.22 ± 0.16^*#^	1.33 ± 0.21	4.50 ± 0.04
G group, %	2.66 ± 0.09	6.66 ± 0.65	0.88 ± 0.23	4.93 ± 0.06^*^
H group, %	4.52 ± 0.16^#&^	5.29 ± 0.33^#^	2.95 ± 1.34	4.16 ± 0.06^&^
In lung tissue, F value	32.898	27.945	53.348	2.291
B group, %	8.94 ± 1.10	0.58 ± 0.00	1.08 ± 0.01	4.03 ± 1.18
C group, %	3.94 ± 0.11	1.33 ± 0.04	0.41 ± 0.09	6.87 ± 1.32
D group, %	3.39 ± 0.32	1.77 ± 0.29	0.11 ± 0.01^*^	8.22 ± 2.21
E group, %	4.94 ± 0.18	0.88 ± 0.06	0.73 ± 0.05	5.14 ± 0.94
F group, %	4.44 ± 0.35	1.24 ± 0.01^*^	0.85 ± 0.16	6.51 ± 1.12
G group, %	3.76 ± 0.01	1.48 ± 0.09	0.28 ± 0.03^*^	7.63 ± 2.29
H group, %	5.29 ± 0.18	0.64 ± 0.01^@%^	0.89 ± 0.01^*#&^	4.21 ± 1.38

Data was expressed as mean ± SD. Compared with B group (NC group), ^*^P < 0.05; compared with C group (DC-SOCS1 I group), ^@^*P* < 0.05; compared with D group (DC-SOCS1 II group), ^#^*P* < 0.05; compared with E group (imDCs I group), ^$^*P* < 0.05; compared with F group (DC-SOCS1 III group), ^%^*P* < 0.05; compared with G group (DC-SOCS1 IV group), ^&^*P* < 0.05

#### Content of mDCs

**Fig. 4:**
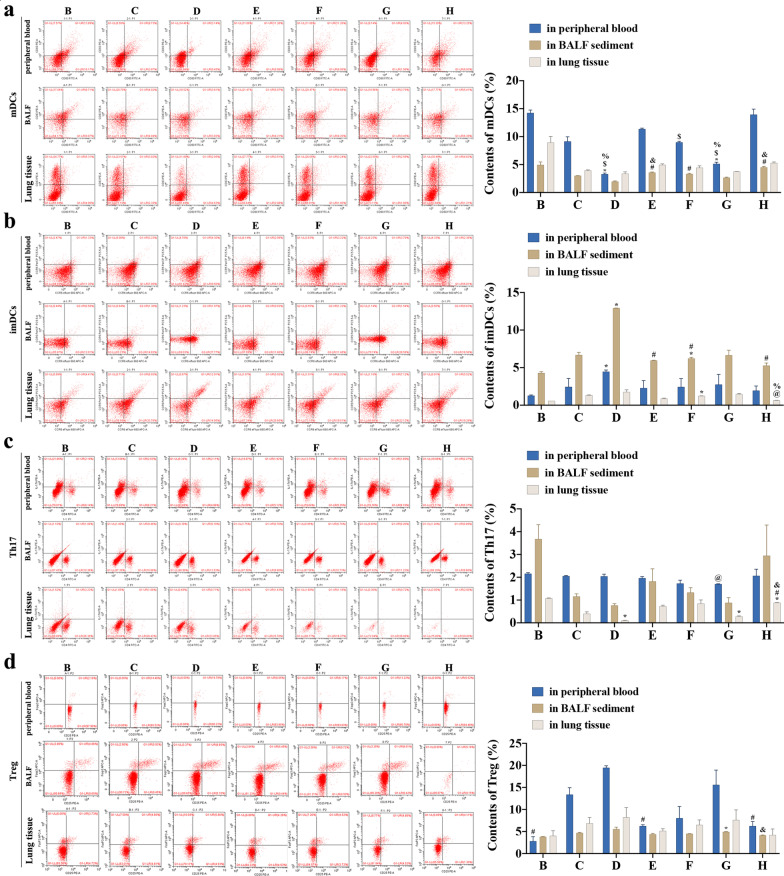
Content of mDCs, imDCs, Th17 and Treg in peripheral blood, BALF sediments and lung tissue of each group. **a** Content of mDCs (CD80^+^CD83^+^) in peripheral blood, BALF sediment, and lung tissue. **b** Content of imDCs (CCR5^+^CCR6^+^) in peripheral blood, BALF sediment, and lung tissue. **c** Content of Th17 (CD4^+^IL-17A^+^) in peripheral blood, BALF sediment, and lung tissue. **d** Content of Treg (CD4^+^CD25^+^Foxp3^+^) in peripheral blood, BALF sediment, and lung tissue. (B: NC group; C: DC-SOCS1 I group; D: DC-SOCS1 II group; E: imDCs I group; F: DC-SOCS1 III group; G: DC-SOCS1 IV group; H: imDCs II group.)

In the peripheral blood of mice, the content of mDCs in B group (14.25 ± 0.50) was significantly higher than that in D (3.29 ± 0.21) and G (5.13 ± 0.28) groups (*P* < 0.05); the mDCs in E group (11.37 ± 0.15) was higher than that in D, F (8.98 ± 0.19) and G groups with statistically significant differences (*P* < 0.05); the mDCs of F group was significantly higher than that of D and G groups (*P* < 0.05). In the BALF sediment, the content of mDCs in D group (1.98 ± 0.11) was lower than that of E (3.58 ± 0.09), F (3.32 ± 0.11) and H (4.52 ± 0.16) groups with significant difference (*P* < 0.05); the content of mDCs of G group (2.66 ± 0.16) was significantly decreased compared with E and H groups (*P* < 0.05). However, no significant differences were found in the content of mDCs in lung tissue between the groups (Fig. 4a).

#### Content of imDCs

The content of imDCs in the peripheral blood of D group (4.46 ± 0.23) was significantly higher than that of B (1.27 ± 0.09) group (*P* < 0.05), while no differences were observed in the content of imDCs in the peripheral blood among the other groups. In the BALF sediment, the content of imDCs of D group (12.92 ± 0.01) was remarkably increased compared with that of E (5.95 ± 0.03), F (6.22 ± 0.16), and H (5.29 ± 0.33) groups (*P* < 0.05); compared with B group (4.92 ± 0.18), the content of imDCs of D and F groups was significantly increased (*P* < 0.05). In addition, the content of imDCs in lung tissue of F group (1.24 ± 0.01) was higher than that of B (0.58 ± 0.00) and H (0.64 ± 0.01) groups (*P* < 0.05), and the content of imDCs in D group (3.39 ± 0.32) was significantly higher than that in H group (*P* < 0.05). No difference was observed in the content of imDCs in BALF sediment and lung tissue among the other groups. (Fig. 4b).

#### Content of Th17

As shown, the content of Th17 of G group (2.75 ± 1.34) in peripheral blood was lower compared with D group (2.05 ± 0.09) (*P* < 0.05). Compared with B group (1.08 ± 0.01) in lung tissue, the content of Th17 of D (0.11 ± 0.01), G (0.28 ± 0.03) and H (0.89 ± 0.01) groups was significantly lower (*P* < 0.05). Additionally, the content of H group in lung tissue was significantly higher than that of D and G (0.28 ± 0.03) groups (*P* < 0.05). No significant differences were observed in Th17 in the BALF sediment between the groups (Fig. 4c).

#### Content of Treg

The content of Treg in peripheral blood of D group (19.47 ± 0.44) was significantly higher than that of B (2.83 ± 1.03), E (6.26 ± 0.29), and H (6.26 ± 1.04) groups (*P* < 0.05), and the content of Treg in BALF sediment of G group (4.93 ± 0.06) was higher than that of B (3.80 ± 0.09) and H (4.16 ± 0.06) groups (*P* < 0.05). However, There was no statistically significant differences were observed in the content of Treg of each group in the lung tissue (Fig. 4d).

### Content of DCs, Th17 and Treg-related cytokines peripheral blood, BALF supernatant, and lung tissue

The expression levels of IL-17, IL-21, IL-23, IL-10, IL-4, IL-5, IL-12, TGF-β and IFN-γ in the BALF supernatant (Table 3) and lung tissue (Table 4) of each group were calculated from the standard curve.

**Table 3:** Contents of DCs, Th17 and Treg cytokines in BALF supernatant of each group

Groups	IL-17, pg/ml	IL-21, ng/l	IL-23, pg/ml	IL-10, pg/ml	TGF-β, ng/l	IFN-γ, ng/l	IL-12, ng/l	IL-4, pg/ml	IL-5, ng/l
F value	1977.027	800.989	3396.573	283.846	2943.466	636.538	1346.904	4868.041	8082.121
A group	/	/	/	/	/	92.48 ± 0.88	19.71 ± 0.47	333.47 ± 2.17	12.77 ± 0.08
B group	78.87 ± 0.91	420.44 ± 6.49	200.62 ± 0.24	39.46 ± 1.12	24.98 ± 0.08	472.45 ± 13.15^+^	40.02 ± 0.29^+^	101.16 ± 1.43^+^	1.07 ± 0.06^+^
C group	34.09 ± 1.24^*^	284.33 ± 2.98^*^	121.09 ± 0.24^*^	58.71 ± 0.77^*^	42.40 ± 0.18^*^	252.28 ± 3.08^+^	30.75 ± 0.05^+*^	238.90 ± 0.90^+*^	6.00 ± 0.04^+*^
D group	24.12 ± 0.20^*^	181.49 ± 6.82^*@^	70.21 ± 0.93^*@^	70.12 ± 0.40^*@^	50.55 ± 0.00^*@^	124.43 ± 0.88^+*@^	25.67 ± 0.31^+*^	304.94 ± 0.82^*@^	10.03 ± 0.08^+*@^
E group	46.46 ± 0.09^*#^	344.41 ± 4.25^*@#^	150.19 ± 0.06^*@#^	50.74 ± 0.98^*#^	36.46 ± 0.48^*#^	328.18 ± 10.08^+*^	35.15 ± 0.01^+@#^	148.89 ± 3.33^+*@#^	3.04 ± 0.02^+*@#^
F group	35.65 ± 0.16^*#$^	307.43 ± 1.04^*^	136.34 ± 0.16^*@#$^	54.78 ± 014^#^	40.12 ± 0.02^*#^	276.23 ± 4.21^+#^	32.59 ± 0.11^+*@#$^	202.95 ± 2.19^+*@#$^	5.38 ± 0.12^+*#^
G group	27.00 ± 0.36^*$%^	226.29 ± 2.47^*@$%^	99.35 ± 2.21^*#$^	62.99 ± 1.59^*^	44.98 ± 0.21^*@#$%^	193.80 ± 10.18^*$^	28.37 ± 0.35^+*^	270.50 ± 0.07^+*@#$%^	8.10 ± 0.00^+*@#$^
H group	55.69 ± 0.17^*#$%&^	378.59 ± 0.23^*@#%&^	167.70 ± 1.36^*@#%&^	44.56 ± 0.15^@#%^	33.27 ± 0.12^*@#%&^	395.25 ± 4.45^+@#%&^	38.58 ± 0.04^+@#$%&^	120.39 ± 0.62^+*@#%&^	2.12 ± 0.01^+@#$%&^

Data was expressed as mean ± SD. Compared with A group (Normal group), ^+^*P* < 0.05; compared with B group (NC group), ^*^P < 0.05; compared with C group (DC-SOCS1 I group), ^@^*P* < 0.05; compared with D group (DC-SOCS1 II group), ^#^*P* < 0.05; compared with E group (imDCs I group), ^$^*P* < 0.05; compared with F group (DC-SOCS1 III group), ^%^*P* < 0.05; compared with G group (DC-SOCS1 IV group), ^&^*P* < 0.05

**Table 4:** Contents of DCs, Th17 and Treg cytokines in lung tissue of each group

Groups	IL-17, pg/ml	IL-21, ng/l	IL-23, pg/ml	IL-10, pg/ml	TGF-β, ng/l	IFN-γ, ng/l	IL-12, ng/l	IL-4, pg/ml	IL-5, ng/l
F value	1524.590	956.826	1103.524	280.963	4455.936	952.512	314.215	5206.059	299.032
A group	/	/	/	/	/	41.53 ± 3.22	19.43 ± 0.03	326.85 ± 0.81	14.42 ± 0.06
B group	52.24 ± 1.16	200.62 ± 0.24	224.10 ± 1.17	35.02 ± 0.54	23.49 ± 0.09	383.00 ± 3.11^+^	40.06 ± 0.05^+^	96.18 ± 0.62^+^	1.53 ± 0.01^+^
C group	13.05 ± 0.46^*^	121.09 ± 0.24^*^	139.88 ± 0.16^*^	54.05 ± 1.77	40.54 ± 0.10^*^	168.58 ± 5.69^+*^	30.74 ± 0.06^+*^	232.99 ± 3.72^+*^	6.12 ± 0.04^+^
D group	6.50 ± 0.53^*@^	70.21 ± 0.93^*@^	92.21 ± 2.52^*^	71.61 ± 1.38^*@^	48.90 ± 0.36^*@^	104.03 ± 7.39^*^	26.07 ± 0.18^+*@^	290.48 ± 0.12^+*^	11.19 ± 3.36
E group	28.12 ± 0.33^*@#^	150.19 ± 0.06^*@#^	176.28 ± 3.92^#^	43.53 ± 0.31^*#^	32.81 ± 0.01^*@#^	217.03 ± 7.67^+*#^	35.13 ± 0.04^+*@#^	144.39 ± 0.04^+*@#^	3.36 ± 0.16^+@^
F group	16.13 ± 0.80^*#$^	136.34 ± 0.16^#^	152.26 ± 1.10^*#^	48.18 ± 0.65^*#^	38.44 ± 0.21^*#$^	195.78 ± 3.50^+*#^	32.63 ± 0.09^+*@#$^	198.23 ± 0.91^+*#$^	5.81 ± 0.04^+*^
G group	8.49 ± 0.12^*$^	99.35 ± 2.21^*$%^	126.30 ± 0.43^*@%^	66.53 ± 1.85^*^	42.23 ± 0.16^*@#$%^	131.78 ± 2.51^+*%^	28.67 ± 0.12^+*@#$%^	260.22 ± 0.19^+*$%^	8.45 ± 0.02^+*@$%^
H group	37.58 ± 0.17^@#$%&^	167.70 ± 1.36^@#%&^	195.42 ± 0.68^*@#%&^	40.00 ± 0.00^#^	30.87 ± 0.04^*@#$%&^	306.95 ± 4.38^+*@#$%&^	38.72 ± 1.51	113.93 ± 1.58^+@#%&^	2.44 ± 0.04^+*@%&^

Data was expressed as mean ± SD. Compared with A group (Normal group), ^+^*P* < 0.05; compared with B group (NC group), ^*^P < 0.05; compared with C group (DC-SOCS1 I group), ^@^*P* < 0.05; compared with D group (DC-SOCS1 II group), ^#^*P* < 0.05; compared with E group (imDCs I group), ^$^*P* < 0.05; compared with F group (DC-SOCS1 III group), ^%^*P* < 0.05; compared with G group (DC-SOCS1 IV group), ^&^*P* < 0.05

#### In BALF supernatant

**Fig. 5:**
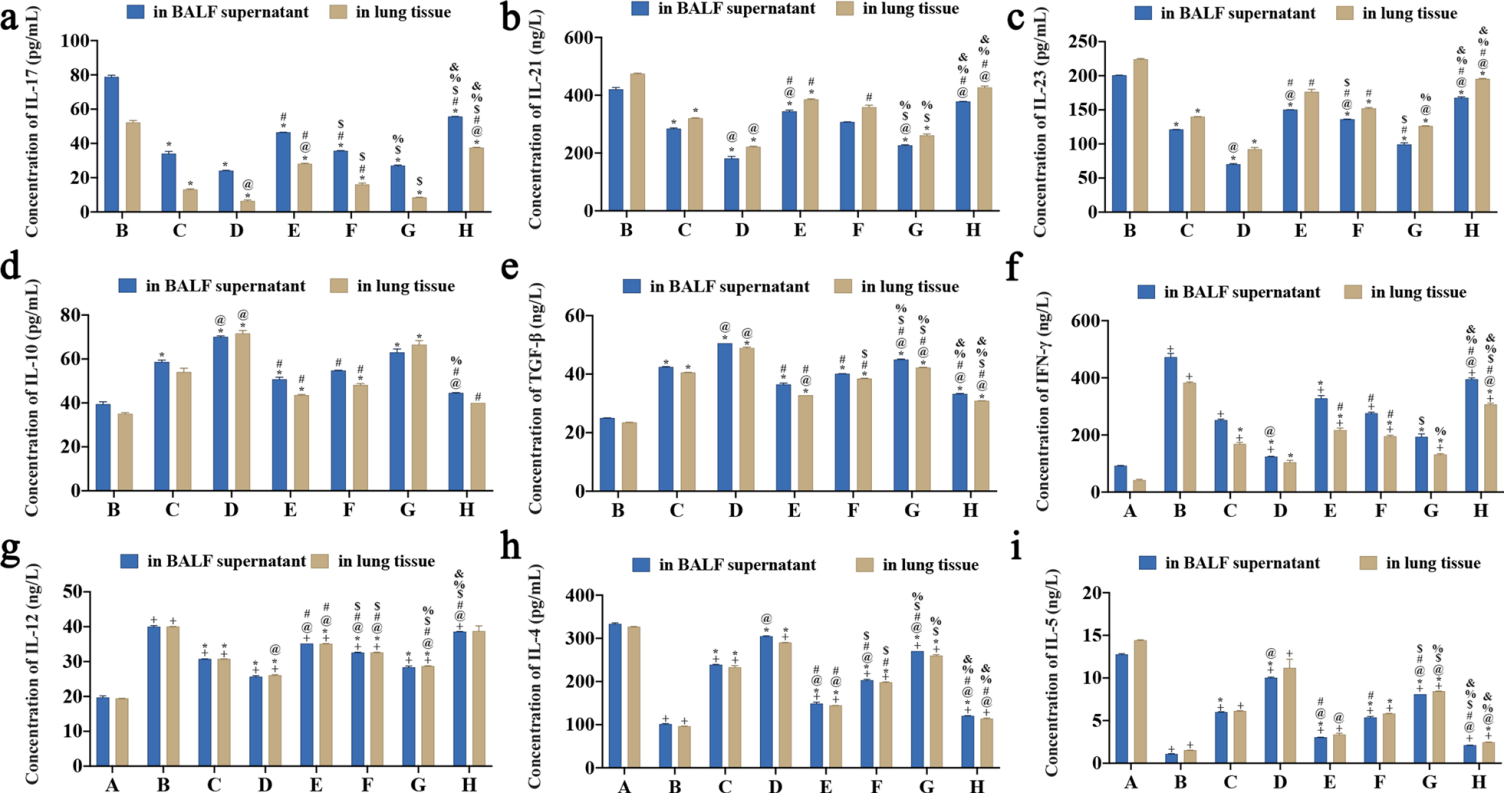
Expression of DCs, Th17 and Treg-related cytokines in BALF supernatant and lung tissue of each group. **a** Expression of IL-17 in BALF supernatant and lung tissue. **b** Expression of IL-21 in BALF supernatant and lung tissue. **c** Expression of IL-23 in BALF supernatant and lung tissue. **d** Expression of IL-10 in BALF supernatant and lung tissue. **e** Expression of TGF-β in BALF supernatant and lung tissue. **f** Expression of IFN-γ in BALF supernatant and lung tissue. **g** Expression of IL-12 in BALF supernatant and lung tissue. **h** Expression of IL-14 in BALF supernatant and lung tissue. **i** Expression of IL-5 in BALF supernatant and lung tissue. (A: Normal group; B: NC group; C: DC-SOCS1 I group; D: DC-SOCS1 II group; E: imDCs I group; F: DC-SOCS1 III group; G: DC-SOCS1 IV group; H: imDCs II group.)

The results showed that the concentration of IL-17 (pg/ml) in C, D, E, F, G, and H groups (34.09 ± 1.24, 24.12 ± 0.20, 46.46 ± 0.09, 35.65 ± 0.16, 27.00 ± 0.36, 55.69 ± 0.17) were significantly lower compared with that of B group (78.87 ± 0.91) (*P* < 0.05); the concentration of IL-17 in E, F and H groups was significantly higher than that of D group (*P* < 0.05); the concentration of IL-17 in E group was significantly higher than in F and G groups, but lower than that in H group (*P* < 0.05); the concentration of IL-17 in F group was lower than in H group but higher than that in G group with statistically significance (*P* < 0.05); the concentration of IL-17 in G group was lower than in H group with statistically significance (*P* < 0.05) (Fig. 5a).

The concentration of IL-21 (ng/l) in C (284.33 ± 2.98), D (181.49 ± 6.82), E (344.41 ± 4.25) and G (226.29 ± 2.47) groups were significantly lower compared with B group (420.44 ± 6.49) (*P* < 0.05); the concentration of IL-21 in C, D, F (307.43 ± 1.04) and G groups were significantly lower than in H group (378.59 ± 0.23) (*P* < 0.05); the concentration of IL-21 in C, E and F groups were increased than that of G group with statistically significance (*P* < 0.05); the concentration of IL-21 in C group was significantly higher than in D group but lower than in E group (*P* < 0.05) (Fig. 5b).

The concentration of IL-23 (pg/ml) in C, D, E, F, G, and H groups (121.09 ± 0.24, 70.21 ± 0.93, 150.19 ± 0.06, 136.34 ± 0.16, 99.35 ± 2.21, 167.70 ± 1.36) were significantly decreased than in B group (200.62 ± 0.24) (*P* < 0.05); the concentration of IL-23 in C, D, F and G groups were significantly lower compared with H group (*P* < 0.05); the concentration of IL-23 in C, D groups were decreased in comparison with E and F groups, and the concentration of IL-23 in F and G groups was lower than that of E group (*P* < 0.05); the concentration of IL-23 in C group was increased than that of D groups with statistically significance (*P* < 0.05) (Fig. 5c).

The concentration of IL-10 (pg/ml) in C (58.71 ± 0.77), D (70.12 ± 0.40), E (50.74 ± 0.98) and G (62.99 ± 1.59) groups were significantly higher compared with B group (39.46 ± 1.12) (*P* < 0.05); the concentration of IL-10 in C group was obviously higher than that in H group but lower than that in D group (*P* < 0.05); the concentration of IL-10 in E, F (54.78 ± 014), and H (44.56 ± 0.15) groups were significantly lower than that of D group (*P* < 0.05); the concentration of IL-10 in F group was significantly increased compared with H group (*P* < 0.05) (Fig. 5d).

The concentration of TGF-β (ng/l) in C, D, E, F, G, and H groups (42.40 ± 0.18, 50.55 ± 0.00, 36.46 ± 0.48, 40.12 ± 0.02, 44.98 ± 0.21, 33.27 ± 0.12) were significantly increased than in B group (24.98 ± 0.08) (*P* < 0.05); the concentration of TGF-β in D group was significantly higher than that of C, E, F, G, H groups (*P* < 0.05); the concentration of TGF-β in C group was significantly higher compared with G, H groups (*P* < 0.05); the concentration of TGF-β in E, H groups were significantly decreased compared with G group (*P* < 0.05); the concentration of TGF-β in F group was significantly higher than that of H group, but lower than that in G group (*P* < 0.05) (Fig. 5e).

In addition, the concentration of IFN-γ (ng/l) in B (472.45 ± 13.15), C (252.28 ± 3.08), D (124.43 ± 0.88), E (328.18 ± 10.08), F (276.23 ± 4.21) and H (395.25 ± 4.45) groups were significantly increased compared with A group (92.48 ± 0.88) (*P* < 0.05); the concentration of IFN-γ in B group was significantly higher than that of D, E (328.18 ± 10.08), and G (193.80 ± 10.18) groups (*P* < 0.05); the concentration of IFN-γ in D group was significantly decreased compared with C, F, H groups (*P* < 0.05); the concentration of IFN-γ in G group was significantly lower than that of E, H groups (*P* < 0.05); the concentration of IFN-γ in H group was significantly higher than that of C, G groups (*P* < 0.05) (Fig. 5f).

The concentration of IL-12 (ng/l) in B (40.02 ± 0.29), C (30.75 ± 0.05), D (25.67 ± 0.31), E (35.15 ± 0.01), F (32.59 ± 0.11), G (28.37 ± 0.35) and H (38.58 ± 0.04) groups were significantly higher than that of A group (19.71 ± 0.47) (*P* < 0.05); the concentration of IL-12 in C, D, F, G groups were significantly lower than that of B group (*P* < 0.05); the concentration of IL-12 in E, F, H groups were significantly higher than in C, D groups (*P* < 0.05); the concentration of IL-12 in E group was significantly higher than that in F group; the concentration of IL-12 in H group was significantly increased in comparison with E, F, G groups (*P* < 0.05) (Fig. 5g).

The concentration of IL-4 (pg/ml) in B (101.16 ± 1.43), C (238.90 ± 0.90), E (148.89 ± 3.33), F (202.95 ± 2.19), G (270.50 ± 0.07) and H (120.39 ± 0.62) groups were significantly lower compared with A group (333.47 ± 2.17) (*P* < 0.05); the concentration of IL-4 in C, D (304.94 ± 0.82), E, F, G, and H groups were significantly increased than that of B group (*P* < 0.05); the concentration of IL-4 in C, E, F, G, H groups were significantly lower than that of D group (*P* < 0.05); the concentration of IL-4 in C group was significantly higher than that of E, F, H groups but lower than that of G group (*P* < 0.05); the concentration of IL-4 in G group was significantly higher than that of E, F, H groups (*P* < 0.05); the concentration of IL-4 in E, H groups were significantly lower than in F group (*P* < 0.05) (Fig. 5h).

The concentration of IL-5 (ng/l) in B, C, D, E, F, G, and H groups (1.07 ± 0.06, 6.00 ± 0.04, 10.03 ± 0.08, 3.04 ± 0.02, 5.38 ± 0.12, 8.10 ± 0.00, 2.12 ± 0.01) were significantly decreased compared with A group (12.77 ± 0.08) (*P* < 0.05); the concentration of IL-5 in B group was significantly decreased than that of C, D, E, F, G groups (*P* < 0.05); the concentration of IL-5 in H group was significantly lower compared with C, D, E, F, G groups (*P* < 0.05); the concentration of IL-5 in E group was significantly decreased than that of C, D, G groups (*P* < 0.05); the concentration of IL-5 in C, F groups were significantly lower than that of D group (*P* < 0.05) (Fig. 5i).

#### In lung tissue

The results showed that the concentration of IL-17 in C, D, E, F, and G groups (13.05 ± 0.46, 6.50 ± 0.53, 28.12 ± 0.33, 16.13 ± 0.80, 8.49 ± 0.12) were significantly lower compared with B group (52.24 ± 1.16) (*P* < 0.05); the concentration of IL-17 in C group was significantly higher than that of D group but lower than that of E, H (37.58 ± 0.17) groups (*P* < 0.05); the concentration of IL-17 in D group was significantly lower than in E, F, H groups (*P* < 0.05); the concentration of IL-17 in E group was lower than in H group with statistically significance (*P* < 0.05); the concentration of IL-17 in E, H groups were significantly higher than that of F, G groups (*P* < 0.05) (Fig. 5a).

The concentration of IL-21 in C (121.09 ± 0.24), D (70.21 ± 0.93), E (150.19 ± 0.06) and G (99.35 ± 2.21) groups were significantly lower compared with B group (200.62 ± 0.24) (*P* < 0.05); the concentration of IL-21 in C, E, F (136.34 ± 0.16), and H (167.70 ± 1.36) groups were significantly higher than in D group (*P* < 0.05); the concentration of IL-21 in E, F, H (167.70 ± 1.36) groups were increased than that of G group with statistically significance (*P* < 0.05); the concentration of IL-21 in F group was significantly lower than in H group; the concentration of IL-21 in C group was significantly lower than in E, H groups (*P* < 0.05) (Fig. 5b).

The concentration of IL-23 in B group (224.10 ± 1.17) were significantly increased than of C (139.88 ± 0.16), D (92.21 ± 2.52), F (152.26 ± 1.10), G (126.30 ± 0.43), and H (195.42 ± 0.68) groups (*P* < 0.05); the concentration of IL-23 in C, D, F, G groups were significantly lower compared with H group (*P* < 0.05); the concentration of IL-23 in C, F groups were increased in comparison with G group with statistically significance (*P* < 0.05); the concentration of IL-23 in D group was lower than that of E (176.28 ± 3.92) and F groups (*P* < 0.05) (Fig. 5c).

The concentration of IL-10 in D (71.61 ± 1.38), E (43.53 ± 0.31), F (48.18 ± 0.65), and G (66.53 ± 1.85) groups were significantly higher compared with B group (35.02 ± 0.54) (*P* < 0.05); the concentration of IL-10 in C (54.05 ± 1.77), E, F, and H (40.00 ± 0.00) groups were significantly lower than that of D group (*P* < 0.05) (Fig. 5d).

The concentration of TGF-β in C, D, E, F, G, and H groups (40.54 ± 0.10, 48.90 ± 0.36, 32.81 ± 0.01, 38.44 ± 0.21, 42.23 ± 0.16, 30.87 ± 0.04) were significantly increased than in B group (23.49 ± 0.09) (*P* < 0.05); the concentration of TGF-β in C group was significantly higher than that of E, H groups but lower than that of D, G groups (*P* < 0.05); the concentration of TGF-β in D group was significantly higher compared with E, F, G, H groups (*P* < 0.05); the concentration of TGF-β in E, F, G groups were significantly increased compared with H group (*P* < 0.05); the concentration of TGF-β in G group was significantly higher than that of E, F groups (*P* < 0.05); the concentration of TGF-β in E group was lower than that of F group with statistically significance (*P* < 0.05) (Fig. 5e).

In addition, the concentration of IFN-γ in B (383.00 ± 3.11), C (168.58 ± 5.69), E (217.03 ± 7.67), F (195.78 ± 3.50), G (131.78 ± 2.51) and H (306.95 ± 4.38) groups were significantly increased compared with A group (41.53 ± 3.22) (*P* < 0.05); the concentration of IFN-γ in B group was significantly higher than that of C, D (104.03 ± 7.39), E, F, G, and H groups (*P* < 0.05); the concentration of IFN-γ in H group was significantly increased compared with C, D, E, F, G groups (*P* < 0.05); the concentration of IFN-γ in D group was significantly lower than that of E and F groups (*P* < 0.05); the concentration of IFN-γ in F group was significantly higher than that of G group (*P* < 0.05) (Fig. 5f).

The concentration of IL-12 in B, C, D, E, F, and G groups (40.06 ± 0.05, 30.74 ± 0.06, 26.07 ± 0.18, 35.13 ± 0.04, 32.63 ± 0.09, 28.67 ± 0.12) were significantly higher than that of A group (19.43 ± 0.03) (*P* < 0.05); the concentration of IL-12 in C, D, E, F, and G groups were significantly lower than that of B group (*P* < 0.05); the concentration of IL-12 in C, E, F, and G groups were significantly higher than in D group (*P* < 0.05); the concentration of IL-12 in C, F, G groups were significantly decreased in comparison with E group (*P* < 0.05); the concentration of IL-12 in F group was higher than that of C, G groups with statistically significance (*P* < 0.05); the concentration of IL-12 in C group was significantly higher compared with G group (*P* < 0.05) (Fig. 5g).

The concentration of IL-4 in B, C, D, E, F, G, and H groups (96.18 ± 0.62, 232.99 ± 3.72, 290.48 ± 0.12, 144.39 ± 0.04, 198.23 ± 0.91, 260.22 ± 0.19, 113.93 ± 1.58) were significantly lower compared with A group (326.85 ± 0.81) (*P* < 0.05); the concentration of IL-4 in C, D, E, F, and G groups were significantly increased than that of B group (*P* < 0.05); the concentration of IL-4 in H groups were significantly lower than that of C, D, F, G groups (*P* < 0.05); the concentration of IL-4 in D group was significantly higher than that of C, E and F groups (*P* < 0.05); the concentration of IL-4 in E group was significantly lower than that of F, G groups (*P* < 0.05); the concentration of IL-4 in F group was significantly lower compared with G group (*P* < 0.05) (Fig. 5h).

The concentration of IL-5 in B (1.53 ± 0.01), C (6.12 ± 0.04), E (3.36 ± 0.16), F (5.81 ± 0.04), G (8.45 ± 0.02), and H (2.44 ± 0.04) groups were significantly decreased compared with A group (14.42 ± 0.06) (*P* < 0.05); the concentration of IL-5 in B group was significantly decreased than that of F, G, and H groups (*P* < 0.05); the concentration of IL-5 in H group was significantly lower compared with C, F, G groups (*P* < 0.05); the concentration of IL-5 in C group was significantly increased compared with E group (*P* < 0.05); the concentration of IL-5 in G group was significantly increased compared with C, E, F groups (*P* < 0.05) (Fig. 5i).

### Morphological differences of COPD models

**Fig. 6:**
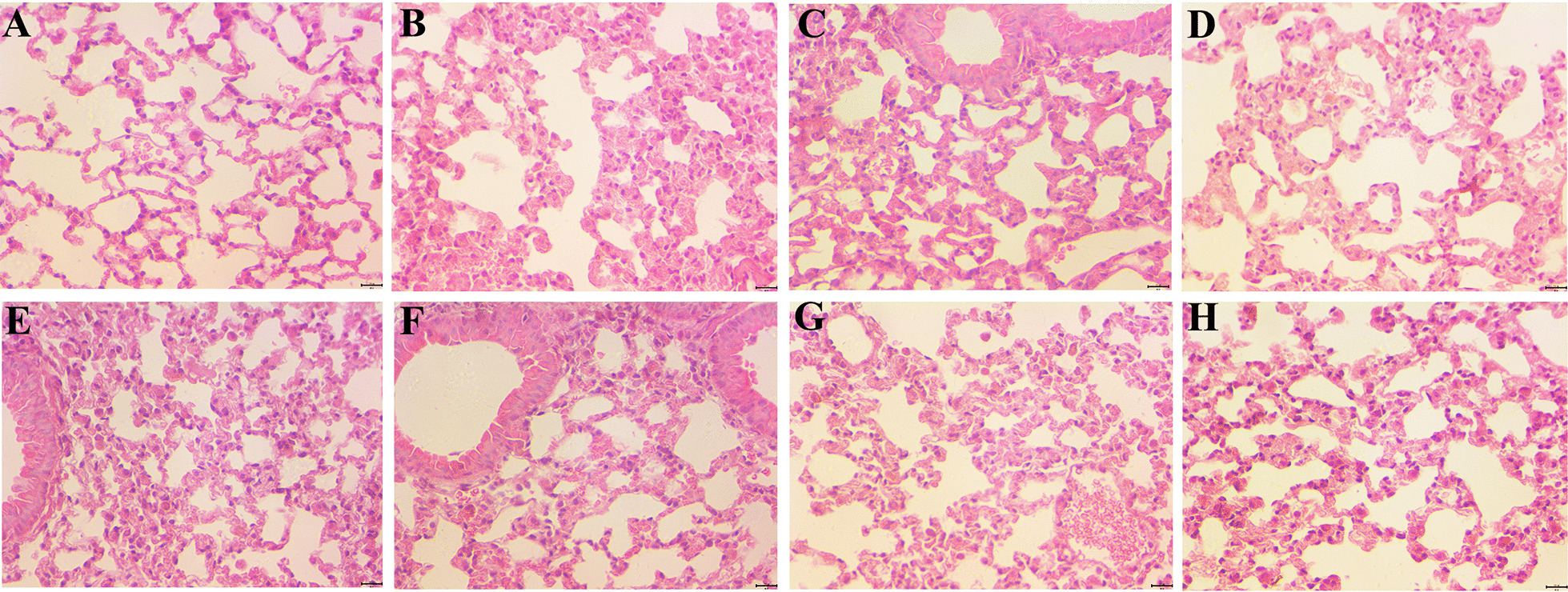
Histopathological changes in the lungs tissue of each group. The histopathological images in the lung tissues of each group (400X). **a** Normal group (n = 3). **b** NC group (n = 5). **c** DC-SOCS1 I group (n = 5). **d** DC-SOCS1 II group (n = 5). **e** imDCs I group (n = 5). **f** DC-SOCS1 III group (n = 5). **g** DC-SOCS1 IV group (n = 5). **h** imDCs II group (n = 5)

Pathological changes in COPD include diffuse dilatation of alveoli, reduction in the number of capillaries in the alveolar walls, narrowing and rupture of alveolar septum, and fusion of enlarged alveoli into larger lumens. As shown in the figure, the pathological changes were significantly aggravated in the B, C, D, E, F, and G groups compared with A group. There were significant changes in the C, D, E, F, and G groups compared with B group, with significant reduction in pathological changes in C, D, F and G groups (Fig. 6).

### Expression of DCs, Th17 and Tregs in lung tissue

**Fig. 7:**
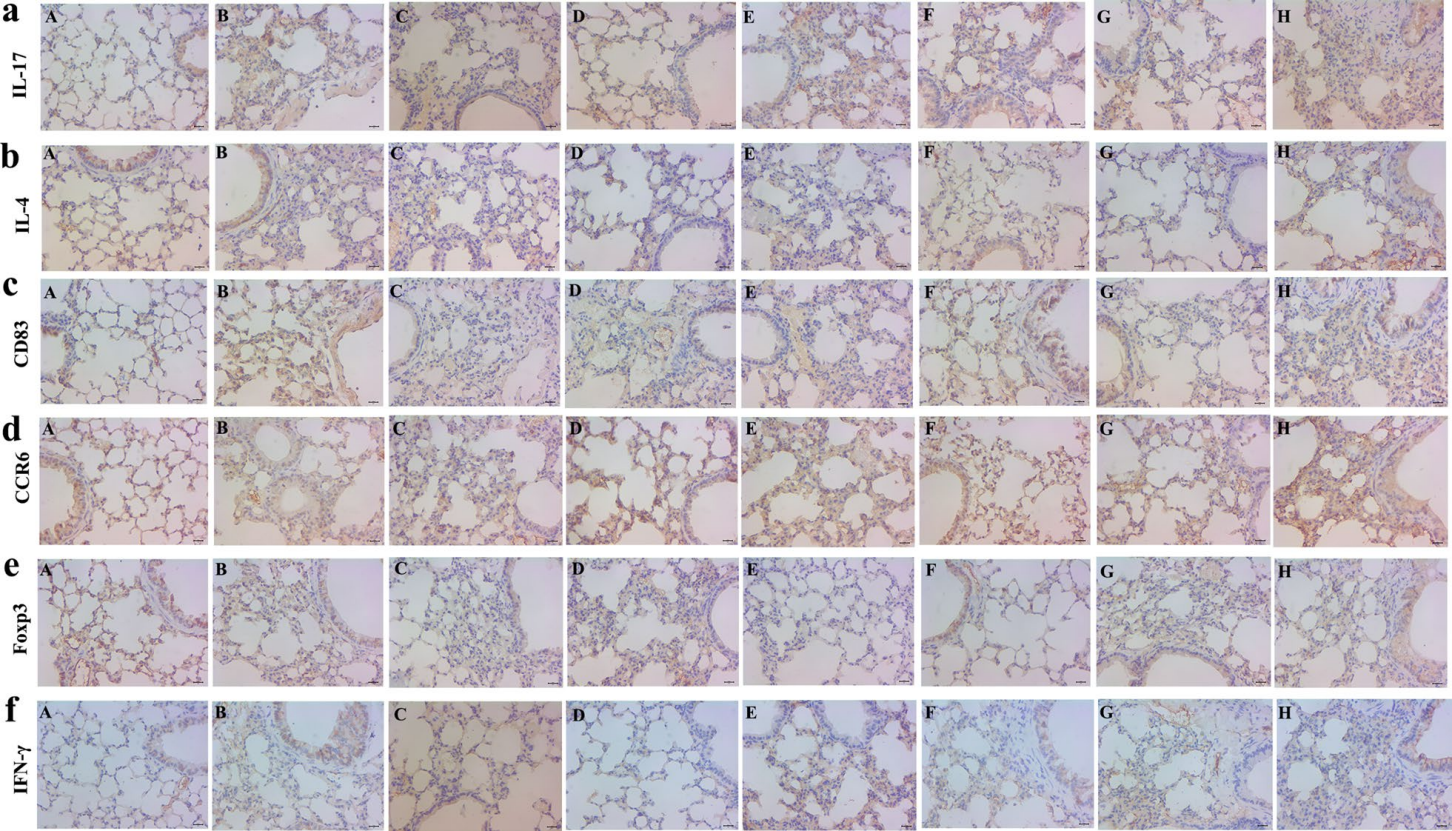
Expression of DCs, Th17 and Treg-related cytokines in lung tissues of each group. **a** Expression of IL-17 in lung tissues. **b** Expression of IL-4 in lung tissues. **c** Expression of CD83 in lung tissues. **d** Expression of CCR6 in lung tissues. **e** Expression of Foxp3 in lung tissues. **f** Expression of INF-γ in lung tissues. (A. Normal group. B. NC group. C. DC-SOCS1 I group. D. DC-SOCS1 II group. E. imDCs I group. F. DC-SOCS1 III group. G. DC-SOCS1 IV group. H. imDCs II group.)

In lung tissues of mice, the expression level of IL-17A, CCR6 and Foxp3 in F, G and H groups was increased, but the expression of CD83, IL-4 and IFN-γ was decreased compared with C, D and E groups (Fig. 7).

## Discussion

COPD is a chronic inflammatory disease of the airways that seriously endangers human health, characterized by a long course, difficult to cure and easy to recur, and characterized by progressive, incomplete and reversible airflow limitation [13]. At present, smoking is considered to be one of the most important pathogenic factors in COPD. Smoking causes chronic inflammation in the airways, and it has been found that 80%-90% of COPD patients are smokers [14]. Chronic inflammation of the airways in COPD patients who had previously smoked was found to persist after fumigation cessation [15].

In this study, we performed smoke stimulation and injected DCs with overexpressed SOCS1 and imDCs through tail vein to observe the lung histopathological manifestations and the changes of inflammatory factors in peripheral blood, BALF and lung tissues in COPD model mice after 4 weeks. The results showed that the lung histopathological manifestations of the mice after 4 weeks of modeling were consistent with those reported in the early literature [16], and were in accordance with the typical pathological features of COPD lung tissues, and the pathological changes in the lung tissues of the mice in the DC-SOCS1 group were significantly reduced compared with those in the control group.

In the present study, the expression of IL-4, Foxp3, and CCR6 was increased in lung tissues of mice reinfused with DC-SOCS1 on day 1 after fumigation compared with mice reinfused with DC-SOCS1 on day 7, but the expression of CD83, IL-17 and IFN-γ was decreased. CD83 is a specific marker of mDCs, and the amount of its expression represents the degree of DC infiltration in tissues [17]. It has been proved that cigarette extracts and nicotine can affect the maturation and function of DCs, and long-term stimulation of DCs with cigarette extracts can lead to a decrease in the expression of CD83. The overexpressed SOCS1 transfected on DCs was able to inhibit DC maturation, and the results showed that the expression level of mDCs in mice reinfused with DC-SOCS1 on day 1 was lower than that on day 7. Additionally, it has been reported that CD83 expression is reduced in the airways of COPD patients and is associated with the expression level of TGF-β, which may inhibit the maturation of DCs as well as CD83 expression [18, 19]. CCR6 is a chemokine receptor expressed on imDCs that regulates the migration of airway imDCs and plays an important role in COPD airway immunity [20]. In addition, it has been demonstrated that the expression of CCR6 and CCL20 in induced sputum was upregulated in COPD patients compared to healthy controls and was significantly correlated with the severity of the disease [21].

Moreover, we observed the changes of DCs, Th17 and Treg in cigarette smoke-induced COPD mouse models in each group, and the results indicated that in peripheral blood, BALF and lung tissues, the content of mDCs and Th17 were higher in the imDCs and DC-SOCS1 groups, and the content of imDCs and Treg were lower compared with the NC group. Previous studies indicated that Th17 can be induced by cigarettes to produce cytokines such as IL-1β and IL-6, which can inhibit Foxp3 expression and Treg differentiation, and to convert Treg to Th17 [22–25]. Daniela et al. found that Th17/Treg cytokine imbalance induced a worsening of the inflammatory process as well as diffuse structural changes in the lungs in a COPD exacerbation model [26]. It suggested that Th17 and Treg mediated immune imbalance contribute to the pathogenesis of COPD.

Th17 has pro-inflammatory activity, and many studies have shown that Th17 and its related cytokines increased significantly in COPD patients and COPD animal models, and enhanced the airway inflammatory response by secreting pro-inflammatory factors such as IL-6 and IL-17, which further led to tissue damage [27–29]. However, Treg could control the inflammatory response of autoimmune diseases by secreting anti-inflammatory factors such as IL-10, IL-35, and TGF-β [30, 31]. Consistent with the results of this study, Wang et al. reported that Treg levels were reduced in COPD patients and animal models of COPD compared to controls [27]. In this study, we found that the expression levels of IL-23, IL-17, IL-12, IL-21 and IFN-γ were significantly decreased in 2 × 10^6^ DC-SOCS1 group than in 1 × 10^6^ DC-SOCS1 and imDCs groups. In particular, the expression levels in 2 × 10^6^ DC-SOCS1 group on day 1 were lower than in 2 × 10^6^ DC-SOCS1 group on day 7. However, the concentrations of IL-10, TGF-β, IL-4, and IL-5 were significantly higher in the 2 × 10^6^ DC-SOCS1 group on day 1 than in the 2 × 10^6^ DC-SOCS1 group on day 7. In addition, the expression levels of IL-10, TGF-β, IL-4 and IL-5 were significantly increased in the group with high concentration of DC-SOCS1 than in the group with low concentration and the imDCs group. IL-17 amplifies the inflammatory response by synergizing with various cytokines [32]. Treg participates in the immune response and maintains homeostasis together with Th17, mainly by secreting anti-inflammatory factors such as IL-10 to suppress inflammatory responses and maintain immune tolerance [33]. SOCS1 can inhibit DCs maturation by suppressing JAK-STAT signaling pathway in DCs cells to maintain DCs stably in the imDCs state [34]. It was found that SOCS1 could inhibit the conversion of Treg cells into Th17 cells [5]. Similarly, in this study, we found that the secretion of pro-inflammatory factors such as IL-17 and IL-23 were decreased and the secretion of anti-inflammatory factors such as IL-10 and TGF-β were increased after injection of DC-SOCS1 compared with the control and imDCs groups. Therefore, the results suggested that DCs transfected with overexpressed SOCS1 could inhibit the secretion of Th17-related cytokines such as IL-17 in COPD.

Moreover, in this study, the content of pro-inflammatory factors such as IL-17 was found to be lower in the 2 × 10^6^ DC-SOCS1 group than in the 1 × 10^6^ DC-SOCS1 group at the same time (day 1 and day 7), while anti-inflammatory factors such as IL-10 and TGF-β increased. The concentrations of pro-inflammatory factors in DCs-SOCS1 injected on day 7 after fumigation were higher than those on day 1, and the concentrations of anti-inflammatory factors decreased. It was suggested that the expression level of inflammatory factors was related to the concentration of injected DCs-SOCS1 and the time of intervention. As IL-10 was involved in the production of regulatory DCs, and overexpression of SOCS1 could induce IL-10 expression on the surface of DCs, so DCs could be converted to regulatory DCs when they produced large amounts of IL-10 [5]. Therefore, the high concentration of DCs-SOCS1 injected on day 1 compared with day 7 resulted in earlier production of regulatory DCs function. As a result, the secretion of anti-inflammatory factors increased and the secretion of pro-inflammatory cytokines such as IL-17 was inhibited, and the increase in IL-10 more effectively contributed to Treg production and earlier protection of Treg function, thus further enhancing the inhibition of inflammation in COPD.

## Conclusions

In summary, early intervention of COPD mice with high concentrations of SOCS1 lentiviral-transfected DCs can reduce the secretion of pro-inflammatory factors in COPD, attenuate the inflammatory response in COPD.

## Supplementary Information

**Additional file 1.** Cover letter.

## Data Availability

The datasets used and/or analysed during the current study available from the corresponding author on reasonable request.

## Abbreviations

APCs: Antigen presenting cells
CD: Cluster of differentiation
COPD: Chronic obstructive pulmonary disease
DCs: Dendritic cells
IL: Interleukin
IFN-γ: Interferon-γ
imDCs: Immaturedendritic cells
mDCs: Mature dendritic cells
SD: Standard deviation
SEM: Standard error of mean
SOCS1: Suppressors of cytokine signaling 1
TGF-β: Transforming growth factor-β
TH17: T helper cell 17
Treg: Regulatory T cell
NC: Negative control

## References

[1] Alcorn JF, Crowe CR, Kolls JK (2010). TH17 cells in asthma and COPD. Annu Rev Physiol.

[2] Vogelmeier CF, Criner GJ, Martinez FJ, Anzueto A, Barnes PJ, Bourbeau J (2017). Global strategy for the diagnosis, management, and prevention of chronic obstructive lung disease 2017 report GOLD executive summary. Am J Respir Crit Care Med..

[3] Cowan AJ, Allen C, Barac A, Basaleem H, Bensenor I, Curado MP (2018). Global burden of multiple myeloma: a systematic analysis for the global burden of disease study 2016. JAMA Oncol.

[4] Roth M (2008). Pathogenesis of COPD, part III: inflammation in COPD. Int J Tuberc Lung Dis..

[5] Takahashi R, Nakatsukasa H, Shiozawa S, Yoshimura A (2017). SOCS1 is a key molecule that prevents regulatory T cell plasticity under inflammatory conditions. J Immunol.

[6] Forsslund H, Mikko M, Karimi R, Grunewald J, Wheelock ÅM, Wahlström J (2014). Distribution of T-cell subsets in BAL fluid of patients with mild to moderate COPD depends on current smoking status and not airway obstruction. Chest.

[7] Steinman RM (2012). Decisions about dendritic cells: past, present, and future. Annu Rev Immunol.

[8] Rowley DA, Fitch FW (2012). The road to the discovery of dendritic cells, a tribute to Ralph Steinman. Cell Immunol.

[9] Tew JG, Wu J, Fakher M, Szakal AK, Qin D (2001). Follicular dendritic cells: beyond the necessity of T-cell help. Trends Immunol.

[10] Merad M, Sathe P, Helft J, Miller J, Mortha A (2013). The dendritic cell lineage: ontogeny and function of dendritic cells and their subsets in the steady state and the inflamed setting. Annu Rev Immunol.

[11] Dong R, Xie L, Zhao K, Zhang Q, Zhou M, He P (2016). Cigarette smoke-induced lung inflammation in COPD mediated via LTB4/BLT1/SOCS1 pathway. Int J Chron Obstruct Pulmon Dis.

[12] Fu H, Song S, Liu F, Ni Z, Tang Y, Shen X (2009). Dendritic cells transduced with SOCS1 gene exhibit regulatory DC properties and prolong allograft survival. Cell Mol Immunol.

[13] Agustí A, Vogelmeier C, Faner R (2020). COPD 2020: changes and challenges. Am J Physiol Lung Cell Mol Physiol.

[14] Kim V, Criner GJ (2013). Chronic bronchitis and chronic obstructive pulmonary disease. Am J Respir Crit Care Med.

[15] Celli BR, MacNee W (2004). Standards for the diagnosis and treatment of patients with COPD: a summary of the ATS/ERS position paper. Eur Respir J.

[16] Vestbo J, Hurd SS, Agustí AG, Jones PW, Vogelmeier C, Anzueto A (2013). Global strategy for the diagnosis, management, and prevention of chronic obstructive pulmonary disease: GOLD executive summary. Am J Respir Crit Care Med.

[17] Zhou LJ, Tedder TF (1995). Human blood dendritic cells selectively express CD83, a member of the immunoglobulin superfamily. J Immunol.

[18] Roghanian A, Drost EM, MacNee W, Howie SE, Sallenave JM (2006). Inflammatory lung secretions inhibit dendritic cell maturation and function via neutrophil elastase. Am J Respir Crit Care Med.

[19] Zanini A, Spanevello A, Baraldo S, Majori M, Della Patrona S, Gumiero F (2014). Decreased maturation of dendritic cells in the central airways of COPD patients is associated with VEGF TGF-β and vascularity. Respiration.

[20] Sun D, Ouyang Y, Gu Y, Liu X (2016). Cigarette smoke-induced chronic obstructive pulmonary disease is attenuated by CCL20-blocker: a rat model. Croat Med J.

[21] Liao SX, Ding T, Rao XM, Sun DS, Sun PP, Wang YJ (2015). Cigarette smoke affects dendritic cell maturation in the small airways of patients with chronic obstructive pulmonary disease. Mol Med Rep.

[22] Saini C, Siddiqui A, Ramesh V, Nath I (2016). Leprosy Reactions Show Increased Th17 Cell Activity and Reduced FOXP3+ Tregs with Concomitant Decrease in TGF-β and Increase in IL-6. PLoS Negl Trop Dis.

[23] Li H, Liu Q, Jiang Y, Zhang Y, Zhang Y, Xiao W (2015). Disruption of th17/treg balance in the sputum of patients with chronic obstructive pulmonary disease. Am J Med Sci.

[24] Josefowicz SZ, Lu LF, Rudensky AY (2012). Regulatory T cells: mechanisms of differentiation and function. Annu Rev Immunol.

[25] Silva LEF, Lourenço JD, Silva KR, Santana FPR, Kohler JB, Moreira AR (2020). Th17/Treg imbalance in COPD development: suppressors of cytokine signaling and signal transducers and activators of transcription proteins. Sci Rep.

[26] Cervilha DAB, Ito JT, Lourenço JD, Olivo CR, Saraiva-Romanholo BM, Volpini RA (2019). The Th17/Treg cytokine imbalance in chronic obstructive pulmonary disease exacerbation in an animal model of cigarette smoke exposure and lipopolysaccharide challenge association. Sci Rep.

[27] Wang H, Ying H, Wang S, Gu X, Weng Y, Peng W (2015). Imbalance of peripheral blood Th17 and Treg responses in patients with chronic obstructive pulmonary disease. Clin Respir J.

[28] Wei B, Sheng LC (2018). Changes in Th1/Th2-producing cytokines during acute exacerbation chronic obstructive pulmonary disease. J Int Med Res.

[29] Long Y, He Y, Jie F, Li S, Wu Y, Li Y (2019). Kuijieling-containing serum regulates Th17 and Treg cell differentiation by inhibiting STAT3 signaling in vitro. Evid Based Complement Alternat Med.

[30] Sakaguchi S, Miyara M, Costantino CM, Hafler DA (2010). FOXP3+ regulatory T cells in the human immune system. Nat Rev Immunol.

[31] Wing JB, Tanaka A, Sakaguchi S (2019). Human FOXP3(+) regulatory T cell heterogeneity and function in autoimmunity and cancer. Immunity.

[32] Rich HE, Alcorn JF (2018). IL-17 strikes a chord in chronic obstructive pulmonary disease exacerbation. Am J Respir Cell Mol Biol.

[33] Stevanin M, Busso N, Chobaz V, Pigni M, Ghassem-Zadeh S, Zhang L (2017). CD11b regulates the Treg/Th17 balance in murine arthritis via IL-6. Eur J Immunol.

[34] Lessard F, Saint-Germain E, Mignacca L, Ferbeyre G (2019). SOCS1: phosphorylation, dimerization and tumor suppression. Oncoscience.

