# Supplementary material for: Effects of SOCS1-overexpressing dendritic cells on Th17- and Treg-related cytokines in COPD mice
Source: BMC Pulm Med. 2022 Apr 15;22:145. doi: 10.1186/s12890-022-01931-1 (PMC9012034; doi:10.1186/s12890-022-01931-1)
Supplement: Supplementary file 1 — Additional file 1. Cover letter. [file 12890_2022_1931_MOESM1_ESM.docx]

38KD

1-1	2-1 3-1 1-2 2-2 3-2	1-3 2-3 3-3

Figure1. B-Actin

38KD

1-1	2-1 3-1 1-2	2-2 3-2	1-3 2-3 3-3

Figure2. SOSC1
